# Modeling of a Magnetoelectric Laminate Ring Using Generalized Hamilton’s Principle

**DOI:** 10.3390/ma12091442

**Published:** 2019-05-03

**Authors:** Ru Zhang, Shengyao Zhang, Yucheng Xu, Lianying Zhou, Futi Liu, Xunqian Xu

**Affiliations:** 1 Department of Civil Engineering, Zhejiang University City College, Hangzhou 310015, China; zhangru@zucc.edu.cn (R.Z.); 31803092@stu.zucc.edu.cn (Y.X.); zhouliany@zucc.edu.cn (L.Z.); 2 Department of Physics, Yibin University, Yibin 644000, China; 2005105005@yibinu.edu.cn (F.L.); 2017105004@yibinu.edu.cn (X.X.)

**Keywords:** magnetoelectric (ME) effect, generalized Hamilton’s principle, laminated composites, magnetostrictive, piezoelectric

## Abstract

The mathematical modeling of the magnetoelectric (ME) effect in ME laminates has been established for some simple structures. However, these methods, which are based on the differential equation approach, are difficult to use in other complex structures (e.g., ring structures). In this work, a new established approach based on the generalized Hamilton’s principle is used to analyze the ME effect in an ME laminated ring. Analytical expressions for ME voltage coefficients are derived. A comparison with the conventional method indicates that this approach is more convenient when the modeling analysis is performed on complex structures. Further, experimental data are also obtained to compare with the theoretical calculations in order to validate the new approach.

## 1. Introduction

The magnetoelectric (ME) effect [1,2], defined as an induced dielectric polarization under the effect of a magnetic field and/or induced magnetization under the action of an electric field, exhibits tremendous potential for applications such as magnetic-field sensors, transducers, energy harvesters, and memory devices [3,4,5]. Extensive research has been and is being performed on ME materials, both experimentally and theoretically [6,7,8,9,10,11,12,13]. Particularly, laminated composites made of magnetostrictive and piezoelectric materials have been emphasized [10,11,12,13]. The ME effects of rectangular laminate, disk–ring, and layered ring–ring composite structures have been studied analytically and experimentally [10,11,12,13,14,15,16,17]. Owing to the strong product effect of the magnetoelastic and elastoelectric effects, they possess large ME effects. Analytical models to explain and predict the ME effect in ME laminate composites should be addressed to provide guidance for the design, fabrication, and application of the ME laminate-based devices. The conventional modeling methods based on differential equations have been established by Bichurin et al. [10,11,12] and Dong et al. [13]. However, these methods were originally proposed for composites with homogeneous material properties. Furthermore, they are applicable to rectangular laminate composites of sandwich structures and are difficult to be implemented for ring structures.

Accordingly, the objective of this study was to investigate a new generalized approach to eliminate these limitations. The generalized Hamilton’s principle [18,19,20,21,22] is proposed, and it can be used to analyze the ME effect in a composite of various laminate structures and/or with inhomogeneous material properties. For comparison, the modeling of an ME laminate ring by the conventional method is first illustrated in Section 2. Subsequently, the modeling process of the ME laminate ring using the new method is presented in Section 3. An experiment is performed to validate the new approach, as detailed in Section 4. It is shown that the proposed method is applicable to cases that can be analyzed by the conventional method, and is convenient for composites with complex laminate structures and inhomogeneous material properties.

## 2. Modeling of ME Laminate Ring by Conventional Method

**Figure 1:**
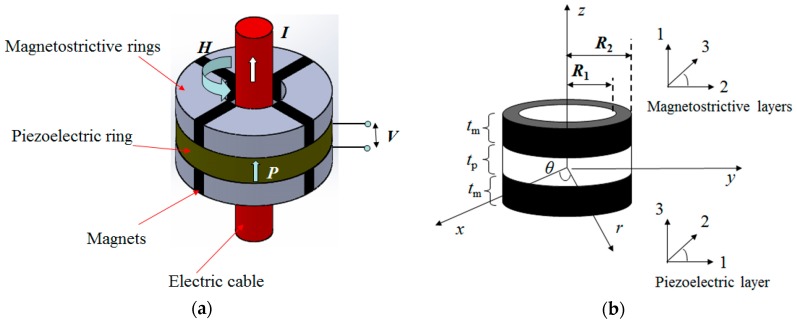
The magnetoelectric (ME) laminated ring: (**a**) An illustration of the configuration. The arrows indicate the directions of AC current (*I*), the polarization (*P*) of the piezoelectric ring, and the magnetic field (*H*); (**b**) The physical model.

Figure 1a illustrates the structure of the ME laminate ring made of a piezoelectric ring sandwiched by two magnetostrictive rings. The magnetostrictive rings are composites of magnetostrictive materials and magnets (used to provide magnetic bias for magnetostriction). The application of a magnetic field by a flow of AC current *I* through the electric cable as shown in Figure 1 will induce a circumferential strain in the magnetostrictive materials that will be subsequently transferred to the piezoelectric material (with polarization along *z* direction) to produce electrical voltage. This process is the principle underlying the ME mechanism in the ME laminate composites. By the conventional modeling method, it is possible to establish the differential governing equation to describe the ME coupling process in the ME laminate ring. Figure 1b shows the physical model of the laminate ring. The cylinder coordinate system (r,θ,z) was used in the modeling process and is shown in Figure 1b. It is noteworthy that the basic assumptions for the model included the following: (1) The magnetostrictive rings were assumed to be homogeneous materials; (2) The deformation mode of the ring is axisymmetric; (3) The bonding between the magnetostrictive and piezoelectric ring is excellent.

Assuming a radial vibration mode, the force equilibrium equation for the laminate ring is

(1) $$2t_{m}^{}\cdot \frac{\partial T_{r,m}^{}}{\partial r}+t_{p}^{}\cdot \frac{\partial T_{r,p}^{}}{\partial r}+2t_{m}^{}\cdot \frac{T_{r,m}^{}-T_{\theta ,m}^{}}{r}+t_{p}^{}\cdot \frac{T_{r,p}^{}-T_{\theta ,p}^{}}{r}=2t_{m}^{}\rho_{m}^{}\cdot \frac{\partial_{}^{2}u_{r}}{\partial t_{}^{2}}+t_{p}^{}\rho_{p}^{}\cdot \frac{\partial_{}^{2}u_{r}}{\partial t_{}^{2}},$$

where $u_{r}^{}$ is the displacement along the radial direction; $\rho_{m}^{}$ and $\rho_{p}^{}$ are the densities for the magnetostrictive and piezoelectric material, respectively; $t_{m}^{}$ and $t_{p}^{}$ are the thicknesses for magnetostrictive and piezoelectric material, respectively; $T_{r,m}$ and $T_{\theta ,m}$ are the stresses in the magnetostrictive material along the radial or circumferential directions, respectively; and $T_{r,p}$ and $T_{\theta ,p}$ are the stress in piezoelectric material along the radial and circumferential directions, respectively. Referring to the constitutive equations of magnetostrictive and piezoelectric materials and the definitions of strains, we derived the governing equation for the ME coupling effect in the laminate ring. The constitutive equations of the magnetostrictive material are:

(2) $$S_{r,m}^{}=s_{11}^{H}T_{r,m}^{}+s_{13}^{H}T_{\theta ,m}^{}+d_{31,m}^{}H_{\theta }^{},\;S_{\theta ,m}^{}=s_{13}^{H}T_{r,m}^{}+s_{33}^{H}T_{\theta ,m}^{}+d_{33,m}^{}H_{\theta }^{},$$

where $s_{ij}^{H}$ (i,j= 1, 2, 3) is the compliance constant; $d_{ij,m}$ (i,j= 1, 2, 3) is the piezomagnetic coefficient; $S_{r,m}$ and $S_{\theta ,m}$ are the strains in the magnetostrictive material along the radial and circumferential directions, respectively; and $H_{\theta }$ is the circumferential component of the magnetic field. The constitutive equations of the piezoelectric material are as follows:

(3) $$S_{r,p}=s_{11}^{E}T_{r,p}+s_{12}^{E}T_{\theta ,p}+d_{31,p}^{}E_{z},\;S_{\theta ,p}=s_{12}^{E}T_{r,p}+s_{11}^{E}T_{\theta ,p}+d_{31,p}^{}E_{z},\;D_{z}^{}=d_{31,p}^{}T_{r,p}^{}+d_{31,p}^{}T_{\theta ,p}^{}+\varepsilon_{33}^{T}E_{z}^{},$$

where $s_{ij}^{E}$ (i,j= 1, 2, 3) is the compliance constant; $d_{ij,p}$ (i,j= 1, 2, 3) is the piezoelectric coefficient; $S_{r,p}$ and $S_{\theta ,p}$ are the strains in the piezoelectric material along the radial and circumferential directions, respectively; $E_{z}$ is the electric field along the axial direction. The material property constants in Equation (2) are dependent on the magnetic bias from the magnets. Note that these material property constants are described in the local coordinate systems for the magnetostrictive and piezoelectric materials as shown in Figure 1, where direction 3 represents the magnetization or polarization direction. For convenience in the following applications, the constitutive equations are reformed as

(4) $$T_{r,m}^{}=c_{11,m}^{}S_{r,m}^{}+c_{13,m}^{}S_{\theta ,m}^{}-e_{31,m}^{}H_{\theta }^{},\;T_{\theta ,m}^{}=c_{13,m}^{}S_{r,m}^{}+c_{33,m}^{}S_{\theta ,m}^{}-e_{33,m}^{}H_{\theta }^{},\;T_{r,p}^{}=c_{11,p}^{}S_{r,p}^{}+c_{12,p}^{}S_{\theta ,p}^{}-e_{31,p}^{}E_{z}^{},\;T_{\theta ,p}^{}=c_{12,p}^{}S_{r,p}^{}+c_{11,p}^{}S_{\theta ,p}^{}-e_{31,p}^{}E_{z}^{},\;D_{z}^{}=e_{31,p}^{}S_{r,p}^{}+e_{31,p}^{}S_{\theta ,p}^{}+\varepsilon_{33}^{}E_{z}^{},$$

where

(5) $$c_{11,m}^{}=\frac{s_{33}^{H}}{s_{11}^{H}s_{33}^{H}-{\left(s_{13}^{H}\right)}^{2}},\;c_{13,m}^{}=\frac{-s_{13}^{H}}{s_{11}^{H}s_{33}^{H}-{\left(s_{13}^{H}\right)}^{2}},c_{33,m}^{}=\frac{s_{11}^{H}}{s_{11}^{H}s_{33}^{H}-{\left(s_{13}^{H}\right)}^{2}},\;\;e_{31,m}^{}=\frac{s_{33}^{H}d_{31,m}^{}-s_{13}^{H}d_{33,m}^{}}{s_{11}^{H}s_{33}^{H}-{\left(s_{13}^{H}\right)}^{2}},e_{33,m}^{}=\frac{-s_{13}^{H}d_{31,m}^{}+s_{11}^{H}d_{33,m}^{}}{s_{11}^{H}s_{33}^{H}-{\left(s_{13}^{H}\right)}^{2}},\;c_{11,p}^{}=\frac{s_{11}^{E}}{{\left(s_{11}^{E}\right)}^{2}-{\left(s_{12}^{E}\right)}^{2}},\;\;c_{12,p}^{}=\frac{-s_{12}^{E}}{{\left(s_{11}^{E}\right)}^{2}-{\left(s_{12}^{E}\right)}^{2}},\;e_{31,p}^{}=\frac{s_{11}^{E}-s_{12}^{E}}{{\left(s_{11}^{E}\right)}^{2}-{\left(s_{12}^{E}\right)}^{2}}\cdot d_{31,p}^{},\;\varepsilon_{33}^{}=\frac{s_{12}^{E}-s_{11}^{E}}{{\left(s_{11}^{E}\right)}^{2}-{\left(s_{12}^{E}\right)}^{2}}\cdot 2d_{31,p}^{2}+\varepsilon_{33}^{T}.$$

The definition of the strains and magnetic field are as follows:

(6) $$S_{r,m}^{}=S_{r,p}^{}=\frac{\partial u_{r}^{}}{\partial r},\;S_{\theta ,m}^{}=S_{\theta ,p}^{}=\frac{u_{r}^{}}{r},\;H_{\theta }=\frac{I}{2\pi r}.$$

By substituting Equations (4)–(6) into Equation (1), we can derive the governing equation for the ME couponing effect in the laminate ring. The equation is expressed as

(7) $$\frac{d_{}^{2}u_{r}}{dr_{}^{2}}+\frac{1}{r}\cdot \frac{du_{r}}{dr}+\left(k_{}^{2}-\frac{v_{}^{2}}{r_{}^{2}}\right)\cdot u_{r}+\frac{\lambda I}{r_{}^{2}}=0,$$

where

(8) $$k_{}^{2}=\frac{\left(2n\rho_{m}^{}+\rho_{p}^{}\right)\omega_{}^{2}}{2nc_{11,m}+c_{11,p}^{}},\;\;v_{}^{2}=\frac{2nc_{33,m}^{}+c_{11,p}^{}}{2nc_{11,m}^{}+c_{11,p}^{}},\;\;\lambda =\frac{1}{\pi }\frac{ne_{33,m}^{}}{2nc_{11,m}^{}+c_{11,p}^{}}.$$

In the equations above, $n=t_{m}/t_{p}$ is the thickness ratio and ω is the angular frequency of the electric current in the electric cable. According to the conventional method, it is necessary to solve the equation above to calculate the ME coefficient in the subsequent step. However, (7) is an inhomogeneous differential equation owing to the inhomogeneous material properties of the laminate ring. Therefore, it is difficult to obtain the analytical solution of the governing equation and calculate the ME coefficient.

## 3. Modeling of ME Laminate Ring by Generalized Hamilton’s Principle

The modeling of the ME laminate ring can be analyzed by the generalized Hamilton’s principle. Hamilton’s principle is a generalized form of the differential equation. The generalized Hamilton’s principle for the laminate ring is expressed as [18,19,20]:

(9) $$\int_{t_{1}^{}}^{t_{2}^{}}\left(\delta K_{}^{*}-\delta U+\delta U_{e}^{*}-\delta W_{e}^{*}\right)dt=0,$$

where *t*_1_ and *t*_2_ are the start and end times, respectively; $\delta K_{}^{*}$ is the variation of the kinetic energy; δU is the variation in mechanical potential energy; $\delta U_{e}^{*}$ is the variation in electric energy; and $\delta W_{e}^{*}$ is the electric work. With the same aforementioned basic assumptions of Section 2, these terms can be expressed as:

(10) $$\delta K_{}^{*}=\int_{\Omega_{m}}^{}\rho_{m}^{}{\dot{u}}_{r}^{}\delta {\dot{u}}_{r}^{}d\Omega +\int_{\Omega_{p}}^{}\rho_{p}^{}{\dot{u}}_{r}^{}\delta {\dot{u}}_{r}^{}d\Omega ,\;\delta U=\int_{\Omega_{m}}^{}\left(T_{r,m}^{}\delta S_{r,m}^{}+T_{\theta ,m}^{}\delta S_{\theta ,m}^{}\right)d\Omega +\int_{\Omega_{p}}^{}\left(T_{r,p}^{}\delta S_{r,p}^{}+T_{\theta ,p}^{}\delta S_{\theta ,p}^{}\right)d\Omega ,\;\;\delta U_{e}^{*}=\int_{\Omega_{p}}^{}D_{z}^{}\delta E_{z}^{}d\Omega ,\;\;\delta W_{e}^{*}=Q\delta V.$$

In the equations above, $\Omega_{m}$ and $\Omega_{p}^{}$ are volumes of the magnetostrictive and piezoelectric materials, respectively; Q and V are the charges applied on the electrodes and voltage of the piezoelectric ring, respectively. For the ME laminate ring, the displacement $u_{r}$ and electric field $E_{z}$ can be written as:

(11a) $$u_{r}^{}\left(r,t\right)=\frac{r-R_{2}^{}}{R_{1}^{}-R_{2}^{}}u_{1}^{}\left(t\right)+\frac{r-R_{1}^{}}{R_{2}^{}-R_{1}^{}}u_{2}^{}\left(t\right),$$

(11b) $$E_{z}\left(t\right)=-\frac{V\left(t\right)}{t_{p}^{}},$$

where $u_{1}^{}$ and $u_{2}^{}$ are the radial displacements at the inner radius $R_{1}^{}$ and outer radius $R_{2}^{}$ of the laminate ring, respectively. Note that the expression above the displacement $u_{r}$ cannot be applied in differential-equation-based methods as it does not satisfy the differential equations. 

By substituting Equations (10), (11), and constitutive equations into Equation (9), the following equations can be obtained:

(12) $$-\omega_{}^{2}M_{11}^{}u_{1}^{}+K_{11}^{}u_{1}^{}-\omega_{}^{2}M_{12}^{}u_{2}^{}+K_{12}^{}u_{2}^{}-N_{1I}^{}I+N_{1V}^{}V=0,\;-\omega_{}^{2}M_{21}^{}u_{1}^{}+K_{21}^{}u_{1}^{}-\omega_{}^{2}M_{22}^{}u_{2}^{}+K_{22}^{}u_{2}^{}-N_{2I}^{}I+N_{2V}^{}V=0,\;N_{1V}^{}u_{1}^{}+N_{2V}^{}u_{2}^{}-C_{V}^{}V=0,$$

where

(13) $$M_{11}^{}=\int_{\Omega_{m}}^{}{\left(\frac{r-R_{2}^{}}{R_{1}^{}-R_{2}^{}}\right)}^{2}\rho_{m}^{}d\Omega +\int_{\Omega_{p}}^{}{\left(\frac{r-R_{2}^{}}{R_{1}^{}-R_{2}^{}}\right)}^{2}\rho_{p}^{}d\Omega ,\;M_{12}^{}=M_{21}=\int_{\Omega_{m}^{}}^{}\frac{\left(r-R_{1}^{}\right)\left(R_{2}^{}-r\right)}{{\left(R_{1}^{}-R_{2}^{}\right)}^{2}}\rho_{m}^{}d\Omega +\int_{\Omega_{p}^{}}^{}\frac{\left(r-R_{1}^{}\right)\left(R_{2}^{}-r\right)}{{\left(R_{1}^{}-R_{2}^{}\right)}^{2}}\rho_{p}^{}d\Omega ,\;M_{22}^{}=\int_{\Omega_{m}^{}}^{}{\left(\frac{r-R_{1}^{}}{R_{2}^{}-R_{1}^{}}\right)}^{2}\rho_{m}^{}d\Omega +\int_{\Omega_{p}^{}}^{}{\left(\frac{r-R_{1}^{}}{R_{2}^{}-R_{1}^{}}\right)}^{2}\rho_{p}^{}d\Omega \;\begin{array}{l} K_{,11}^{}=\int_{\Omega_{m}}^{}\frac{1}{{\left(R_{1}^{}-R_{2}^{}\right)}^{2}}c_{11,m}^{}d\Omega +2\int_{\Omega_{m}}^{}\frac{r-R_{2}^{}}{r{\left(R_{1}^{}-R_{2}^{}\right)}^{2}}c_{13,m}^{}d\Omega +\int_{\Omega_{m}}^{}{\left(\frac{r-R_{2}^{}}{r(R_{1}^{}-R_{2}^{})}\right)}^{2}c_{33,m}^{}d\Omega \\ +\int_{\Omega_{p}}^{}\frac{1}{{\left(R_{1}^{}-R_{2}^{}\right)}^{2}}c_{11,p}^{}d\Omega +2\int_{\Omega_{p}}^{}\frac{r-R_{2}^{}}{r{\left(R_{1}^{}-R_{2}^{}\right)}^{2}}c_{12,p}^{}d\Omega +\int_{\Omega_{p}}^{}{\left(\frac{r-R_{2}^{}}{r(R_{1}^{}-R_{2}^{})}\right)}^{2}c_{11,p}^{}d\Omega \end{array}\;\begin{array}{l} K_{12}^{}=K_{21}^{}=-\int_{\Omega_{m}^{}}^{}\frac{1}{{\left(R_{1}^{}-R_{2}^{}\right)}^{2}}c_{11,m}^{}d\Omega +\int_{\Omega_{m}^{}}^{}\frac{R_{1}^{}+R_{2}^{}-2r}{r{\left(R_{1}^{}-R_{2}^{}\right)}^{2}}c_{13,m}^{}d\Omega +\int_{\Omega_{m}^{}}^{}\frac{\left(r-R_{1}^{})(R_{2}^{}-r\right)}{r^{2}{\left(R_{1}^{}-R_{2}^{}\right)}^{2}}c_{33,m}^{}d\Omega \\ -\int_{\Omega_{p}^{}}^{}\frac{1}{{\left(R_{1}^{}-R_{2}^{}\right)}^{2}}c_{11,p}^{}d\Omega +\int_{\Omega_{p}^{}}^{}\frac{R_{1}^{}+R_{2}^{}-2r}{r{\left(R_{1}^{}-R_{2}^{}\right)}^{2}}c_{12,p}^{}d\Omega +\int_{\Omega_{p}^{}}^{}\frac{\left(r-R_{1}^{})(R_{2}^{}-r\right)}{r^{2}{\left(R_{1}^{}-R_{2}^{}\right)}^{2}}c_{11,p}^{}d\Omega \end{array}\;\begin{array}{l} K_{22}^{}=\int_{\Omega_{m}}^{}\frac{1}{{\left(R_{2}^{}-R_{1}^{}\right)}^{2}}c_{11,m}^{}d\Omega +2\int_{\Omega_{m}}^{}\frac{r-R_{1}^{}}{r{\left(R_{2}^{}-R_{1}^{}\right)}^{2}}c_{13,m}^{}d\Omega +\int_{\Omega_{m}}^{}{\left(\frac{r-R_{1}^{}}{r(R_{2}^{}-R_{1}^{})}\right)}^{2}c_{33,m}^{}d\Omega \\ +\int_{\Omega_{p}^{}}^{}\frac{1}{{\left(R_{2}^{}-R_{1}^{}\right)}^{2}}c_{11,p}^{}d\Omega +2\int_{\Omega_{p}^{}}^{}\frac{r-R_{1}^{}}{r{\left(R_{2}^{}-R_{1}^{}\right)}^{2}}c_{12,p}^{}d\Omega +\int_{\Omega_{p}^{}}^{}{\left(\frac{r-R_{1}^{}}{r(R_{2}^{}-R_{1}^{})}\right)}^{2}c_{11,p}^{}d\Omega \end{array}\;N_{1V}^{}=\int_{\Omega_{p}}^{}e_{31,p}^{*}\frac{1}{t_{p}^{}(R_{1}^{}-R_{2}^{})}d\Omega +\int_{\Omega_{p}}^{}e_{31,p}^{*}\frac{r-R_{2}^{}}{t_{p}^{}r(R_{1}^{}-R_{2}^{})}d\Omega ,\;N_{2V}^{}=\int_{\Omega_{p}}^{}e_{31,p}^{*}\frac{1}{t_{p}^{}(R_{2}^{}-R_{1}^{})}d\Omega +\int_{\Omega_{p}}^{}e_{31,p}^{*}\frac{r-R_{1}^{}}{t_{p}^{}r(R_{2}^{}-R_{1}^{})}d\Omega ,\;N_{1I}^{}=\int_{\Omega_{m}}^{}e_{31,m}^{*}\frac{1}{2\pi r(R_{1}^{}-R_{2}^{})}d\Omega +\int_{\Omega_{m}}^{}e_{33,m}^{*}\frac{r-R_{2}^{}}{2\pi r^{2}(R_{1}^{}-R_{2}^{})}d\Omega ,\;N_{2I}^{}=\int_{\Omega_{m}}^{}e_{31,m}^{*}\frac{1}{2\pi r(R_{2}^{}-R_{1}^{})}d\Omega +\int_{\Omega_{m}}^{}e_{33,m}^{*}\frac{r-R_{1}^{}}{2\pi r^{2}(R_{2}^{}-R_{1}^{})}d\Omega ,\;C_{V}^{}=\int_{\Omega_{p}}^{}\frac{\varepsilon_{33}^{}}{t_{p}^{2}}d\Omega .$$

Equation (12) can be solved more easily than Equation (7) because it is a group of linear algebraic equations. The ME voltage coefficient $\alpha_{V}$ of the ME laminate ring can be solved from Equation (12) as follows:

(14) $$\alpha_{V}=\left|\frac{dV}{dI}\right|=\left|\frac{\frac{N_{1V}^{}\left[N_{1I}^{}\left(-\omega_{}^{2}M_{22}^{}+K_{22}^{}\right)-N_{2I}^{}\left(-\omega_{}^{2}M_{12}^{}+K_{12}^{}\right)\right]+N_{2V}^{}\left[-N_{1I}^{}\left(-\omega_{}^{2}M_{21}^{}+K_{21}^{}\right)+N_{2I}^{}\left(-\omega_{}^{2}M_{11}^{}+K_{11}^{}\right)\right]}{\left(-\omega_{}^{2}M_{11}^{}+K_{11}^{}\right)\left(-\omega_{}^{2}M_{22}^{}+K_{22}^{}\right)-\left(-\omega_{}^{2}M_{21}^{}+K_{21}^{}\right)\left(-\omega_{}^{2}M_{12}^{}+K_{12}^{}\right)}}{C_{V}^{}-\frac{N_{1V}^{}\left[-N_{1V}^{}\left(-\omega_{}^{2}M_{22}^{}+K_{22}^{}\right)+N_{2V}^{}\left(-\omega_{}^{2}M_{12}^{}+K_{12}^{}\right)\right]+N_{2V}^{}\left[N_{1V}^{}\left(-\omega_{}^{2}M_{21}^{}+K_{21}^{}\right)-N_{2V}^{}\left(-\omega_{}^{2}M_{11}^{}+K_{11}^{}\right)\right]}{\left(-\omega_{}^{2}M_{11}^{}+K_{11}^{}\right)\left(-\omega_{}^{2}M_{22}^{}+K_{22}^{}\right)-\left(-\omega_{}^{2}M_{21}^{}+K_{21}^{}\right)\left(-\omega_{}^{2}M_{12}^{}+K_{12}^{}\right)}}\right|.$$

For convenience, Equation (14) can be simplified as:

(15) $$\alpha_{V}=\left|\frac{1/K_{eff}^{}}{1-\frac{\omega_{}^{2}}{\omega_{0}^{2}}+j2\xi \frac{\omega }{\omega_{0}^{}}}\right|,$$

where ξ is the factor to account for the damping effect in ME coupling, and

(16) $$\omega_{0}^{}=\sqrt{\frac{K_{eff}^{}}{M_{eff}^{}}},\;M_{eff}^{}=\frac{C_{V}^{}\left(M_{11}^{}K_{22}^{}+M_{22}^{}K_{11}^{}-M_{21}^{}K_{12}^{}-M_{12}^{}K_{21}^{}\right)}{N_{1V}^{}\left(N_{1I}^{}K_{22}^{}-N_{2I}^{}K_{12}^{}\right)+N_{2V}^{}\left(-N_{1I}^{}K_{21}^{}+N_{2I}^{}K_{11}^{}\right)},\;K_{eff}^{}=\frac{C_{V}^{}\left(K_{11}^{}K_{22}^{}-K_{12}^{}K_{21}^{}\right)-\left[N_{1V}^{}\left(-N_{1V}^{}K_{22}^{}+N_{2V}^{}K_{12}^{}\right)+N_{2V}^{}\left(N_{1V}^{}K_{21}^{}-N_{2V}^{}K_{11}^{}\right)\right]}{N_{1V}^{}\left(N_{1I}^{}K_{22}^{}-N_{2I}^{}K_{12}^{}\right)+N_{2V}^{}\left(-N_{1I}^{}K_{21}^{}+N_{2I}^{}K_{11}^{}\right)}.$$

## 4. Experimental Procedure

**Figure 2:**
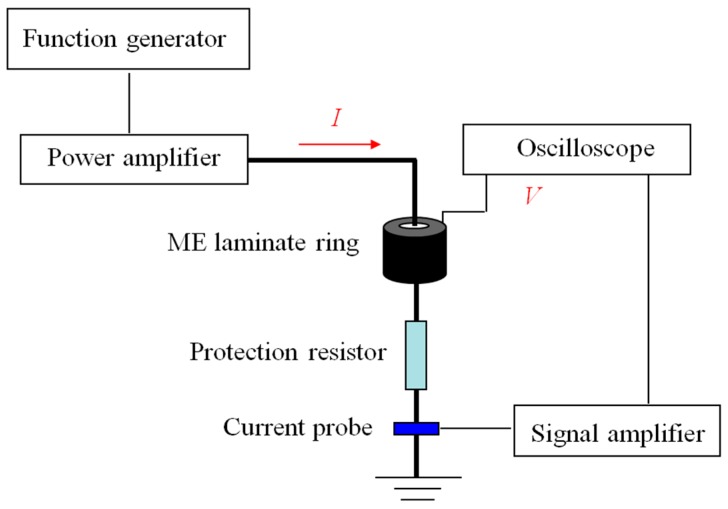
A schematic diagram of the experimental setup.

To verify the new method, a sample of the laminate ring with dimensions $R_{1}^{}$ = 5 mm, $R_{2}^{}=$ 12.5 mm, and $t_{m}=t_{p}=$ 3 mm was fabricated and tested in our laboratory. Terfenol-D/epoxy composites and NdFeB magnets were used to fabricate the magnetostrictive rings, and Pb(Zr,Ti)O_3_ was used to create the piezoelectric ring. The sandwich structure was laminated with silver-loaded epoxy and cured under high pressure at room temperature for over 24 h. The material parameters for calculation were according to the datasheet from the manufacturer and Refs. [23,24,25]. A schematic diagram of the experimental setup is shown in Figure 2. A function generator (Agilent AFG3022B) amplified by a power amplifier (AE Techron TEC7572, AE Techron, Elkhart, IN, USA) was used to generate an AC current in an electric cable to produce an AC magnetic field during testing. According to Ampere’s law, the magnetic field was not uniformly distributed in the laminate ring. The magnetic field reached the maximum or minimum value at the inner or outer radius of the ring, respectively. The waveform of the output voltage from the ME laminate ring was recorded on an oscilloscope (Tektronix DPO2014, Tektronix, OR, USA). The AC current was monitored by a current probe (Hioki 9273&3271, Hioki, Nagano, Japan). Because the magnets in the magnetostrictive rings mentioned previously provided the magnetic bias field, the external DC magnetic field was not necessary in this test. In this testing, the peak-to-peak value of the input current $I_{\mathrm{pk}-\mathrm{pk}}$ of the electric cable was maintained at a constant value of 1 A, and the peak-to-peak value of the corresponding output voltage $V_{\mathrm{pk}-\mathrm{pk}}$ of the ME laminate ring was recorded for different frequencies, which were tuned in the function generator. The ratios of $V_{\mathrm{pk}-\mathrm{pk}}$ and $I_{\mathrm{pk}-\mathrm{pk}}$ provided the measured ME voltage coefficient *α_V_* of the laminate ring. The results indicated that *α_V_* was a function of the AC-current frequency. 

## 5. Results and Discussion

**Figure 3:**
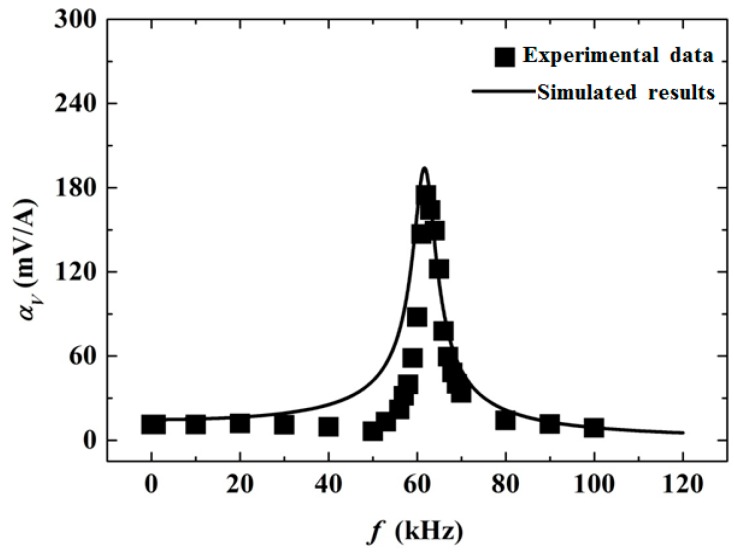
A comparison of the results of the theoretical calculations from the proposed method and the experimental measurements for the ME laminate ring.

Figure 3 shows a comparison of the theoretical calculations from the generalized Hamilton’s principle and experimental measurements for the frequency response of the ME voltage coefficient $\alpha_{V}$ of the laminate ring. The solid line represents the modeling results using Equation (14). It is shown that the ME voltage coefficient $\alpha_{V}$ was almost constant in the low-frequency range with a value of 15 mV/A. As the frequency increased, $\alpha_{V}$ became larger, and near the frequency of 62 kHz, it reached a value as high as 200 mV/A. With a further increase in the frequency, $\alpha_{V}$ decreased quickly. This phenomenon is the typical resonance ME effect in ME composites. From the established model, it is shown that the ME resonance effect in the laminates was caused by vibration resonance, as the ME effect in the laminates was realized by mechanical coupling. The experimental results, shown in rectangles, showed satisfactory agreement with the theoretical results. The small discrepancy in the theoretical curve with experimental data might be introduced by the following factors. First, as shown in Figure 1, an ideal model where the magnetostrictive rings were assumed as homogeneous materials without considering the permanent magnets was established for the ME laminate ring. Next, a perfect bonding between the layers was assumed, implying that the strain of the magnetostrictive materials transferred to the piezoelectric material without loss. In fact, the unpredictable stress and strain introduced by the elasticity and viscosity of the bonding materials may have decreased the strain transfer efficiency and significantly affected the errors between the model and experiment [14,15,16]. Subsequently, an axisymmetric deformation was assumed in the theory where only the normal strain was considered and the shear strain was neglected. However, overall, the generalized Hamilton’s principle provides a convenient and effective method to analyze the ME effect in the ME laminate ring. 

## 6. Conclusions

The modeling of the ME coupling effect in a laminate ring was presented herein using a more generalized theory based on the generalized Hamilton’s principle, in which the ME effect in ME composites could be described by a group of algebraic equations rather than by differential equations as in conventional ME modeling methods. It was shown that this proposed method was more convenient than the conventional methods for complex ME structures owing to the exemption from complex solution methods required for differential equations. The agreement observed between the experimental measurements and theoretical calculations validated the analysis of the laminate ring by the new modeling method. Therefore, the generalized Hamilton’s principle provided an alternative approach that was more simple and convenient to analyze the ME effect in ME composites with complex structures and inhomogeneous material properties.
